# The WHO initiative to measure the effectiveness and impact of PHSM – key activities in 2022

**DOI:** 10.1093/eurpub/ckac129.533

**Published:** 2022-10-25

**Authors:** R Ludolph, R Takahashi, T Nguyen, S Briand

**Affiliations:** High Impact Events Preparedness Unit, WHO, Geneva, Switzerland

## Abstract

The presentation will focus on two main outcomes of the WHO initiative: a global research agenda to steer future evidence generation on PHSM, and a central monitoring system for PHSM research. In September 2021, a global technical consultation with over 60 global experts was organized to review the existing evidence on PHSM and identify the initiative's priorities. The consultation provided an opportunity to have an initial discussion on potential research priorities. This became the basis for an iterative online consultation process. The draft research agenda includes seven main research themes including effectiveness, unintended consequences, methodological challenges and implementation considerations affecting the uptake of and adherence to PHSM. Workshop participants will be invited to comment on the suggested themes and propose additional priority questions for the research agenda. The central research monitoring system will consist of a global repository of primary studies and reviews investigating the effectiveness and broader multisectoral impact of PHSM. Indexed studies will be mapped against the key themes of the research agenda, facilitating real-time monitoring and evaluation of its progress. An AI-based mechanism for automated updating of systematic reviews will complement the database. This one-stop shop will allow researchers and decision-makers worldwide to access the latest evidence on PHSM and keep track of the synthesized effectiveness and impact of different interventions and combinations. The platform will further provide a protected working interface. This monitoring system for PHSM research enables timely access to and utilization of evidence indecision-making processes during health emergencies and fosters international collaboration on the analysis and interpretation of data. Workshop participants will be invited to review the alpha version of the platform.

